# Outcomes of Computer-Assisted Total Knee Arthroplasty Compared to Conventional TKA: A Bicentric Controlled Retrospective Clinical Study

**DOI:** 10.3390/jcm10153352

**Published:** 2021-07-29

**Authors:** Biagio Zampogna, Stefano Campi, Guglielmo Torre, Eleonora Villari, Francesco Moncada, Aristide Perrino, Letterio Ciriaco, Marco Ferlazzo, Rocco Papalia, Vincenzo Denaro

**Affiliations:** 1 Department of Orthopedics and Trauma Surgery, Campus Bio-Medico University of Rome, Via Álvaro del Portillo 200, 00128 Rome, Italy; b.zampogna@unicampus.it (B.Z.); s.campi@unicampus.it (S.C.); eleonora.villari@hotmail.it (E.V.); f.moncada@unicampus.it (F.M.); a.perrino@unicampus.it (A.P.); r.papalia@unicampus.it (R.P.); denaro@unicampus.it (V.D.); 2 Multi-Specialist Clinical Institute for Orthopaedic Trauma Care (COT), 98124 Messina, Italy; letteriociriaco@gmail.com (L.C.); mferlazzo@cotmessina.it (M.F.)

**Keywords:** Total Knee Arthroplasty, knee, computer assisted surgery, navigation system

## Abstract

Despite the globally ascertained success of Total Knee Arthroplasty (TKA) procedure, 20% of patients are still unsatisfied with the surgery results. The purpose of the study is to identify the functional and radiological outcomes of the computer-assisted (CAS) TKA compared to the conventional technique. The clinical databases and medical records of both clinical sites were retrospectively analyzed, and then according to study time-lapse, inclusion, and exclusion criteria, eligible patients were retrieved and included. A total of 42 patients that underwent to CAS TKA (NAVI) and 61 patients that underwent to Conventional TKA (CONV) were included. The NAVI group reported a statistically significant higher surgical time. A lower intraoperative blood loss was found in the computer-assisted group, though this difference was not statistically significant. Implant survival analysis at two years did not show differences between groups during the follow-up period. At two years, follow-up postoperative intergroup analysis showed no statistically significant difference between groups. According to the radiologic analysis, the NAVI group showed comparable outcomes to the conventional group. The present study showed that there was no clinical and radiological difference between CAS arthroplasty and conventional technique.

## 1. Introduction

Over the past few decades, TKA has been considered an efficient solution to alleviate pain and restore physical function in patients with severe osteoarthritis, thus TKA is an increasingly widespread procedure in orthopedic surgical practice [1]. Despite the TKA procedure is globally successful in restoring knee function and resolve symptoms, the 20% of patients are still unsatisfied after surgery [2]. In a recent study, Mahdi et al. reported that out of 17.2% of unhappy patients one year after surgery, only 2% had a perioperative complication. Therefore, 15.2% of patients were unsatisfied even without experiencing any surgical or medical complication [3]. To identify the correct component positioning, the use of radiological (preoperative) and anatomical (intraoperative) measurements is mandatory. To reduce the risk of revision due to malalignment, it is essential to achieve a correct mechanical alignment and an accurate kinematic of the replaced joint [4]. Computer-assisted navigation has been introduced to help surgeons to achieve a more accurate component positioning. This technology is based on sensors capable of detecting the components positioning relatively to specific bone landmarks. Currently, only a few studies investigated navigation in orthopedic surgery without leading to definite conclusions. In most cases, the follow-up is too short due to the recent introduction of these systems. Some recent studies have shown significant advantages (less deviation of the mechanical axis on the coronal plane) in a very short period [5]. However, functional results showed no significant difference between patients treated with navigation and those treated with the traditional technique, neither in the short (1–2 years) nor the long term (5–7 years) [6]. Among the perioperative parameters, it was observed that the intraoperative blood loss was significantly lower in patients operated with navigation-assisted technique compared to those operated with a traditional technique [7]. The purpose of the present study is to investigate functional recovery after navigated and conventional TKA and to examine the radiologically-assessed component alignment. The main hypothesis underlying the investigation is that these outcomes are comparable for both the techniques.

## 2. Materials and Methods

This is a bicentric comparative retrospective clinical study on outcomes of computer-assisted TKA compared to conventional TKA. The study received approval from the local ethics committee (Prot: 53/19 OSS ComEt CBM). Involved study centers are the Department of Orthopedics and Trauma Surgery of Campus Bio-Medico University Hospital (Rome, Italy) and Orthopedics Unit of Multi-Specialist Clinical Institute for Orthopaedic Trauma Care (COT, Messina, Italy).

### 2.1. Study Design

Clinical databases and medical records of both centers were retrospectively analyzed, including patients for evaluation, according to inclusion and exclusion criteria. The study population was composed of patients who underwent TKA from January 2017 to December 2018. One experienced surgeon for each investigational site performed all surgical procedures. Only patients with the one type of implant (Exactech Optetrak Logic Primary Knee System) for both techniques and one navigation system (Exactech GPS^®^ Guided Personalized Surgery) for the study group were included for evaluation. Were excluded from the study patients affected by inflammatory arthritis, post-traumatic OA secondary knee OA, homolateral hip and ankle OA, tumors, rheumatological pathology, psychiatric illness, and history of alcohol abuse or drug abuse.

### 2.2. Surgical Technique with CAS TKA

The navigation system used is Exactech GPS^®^ (Guided Personalized Surgery—Exactech, Gainesville, FL, USA). This is a passive, image-free system, and it is based on intraoperative data collection about joint kinematics and anatomy. A compact screen composes the system with a built-in optical camera easily accessible to the surgeon, one probe for patient data acquisitions, three active infrared trackers (one for tibia reference, one for femur reference, and one for instrument reference). These instruments allow the system to record the relative leg position into space. The procedure follows the same surgical steps as the traditional one until the joint’s total exposure. Then, the calibration procedure needs to be executed by fixing two trackers to the bone to act as a fixed reference for the femur and tibia. Bony landmarks are acquired through a pointer, then the joint stability, the valgus-varus angle, and the tibial slope values are assessed. The bone cuts are then ready to be performed, positioning the cutting guide according to GPS guidance. The surgeon can still check the guide’s position, identify the cutting lines, and eventually modify parameters based on their clinical and technical judgment. The system returns the following data at the end of femoral and tibial preparation: varus-valgus angle, medial and lateral femorotibial space in flexion and extension, femoral rotation, and component position. The intervention is completed as for the procedure with traditional instrumentation. At the end of the procedure, a final report is extracted and downloaded.

### 2.3. Perioperative Evaluation

Following data were extracted from institutional medical records and surgical reports: Age, sex, height, weight, Body Mass Index (BMI), comorbidity, ASA score, date of surgery, affected knee, follow-up length, surgical time, intraoperative blood loss, length of hospital stays and perioperative complications.

### 2.4. Functional Evaluation

The electronic medical records of both the clinical sites were queried to retrieve the functional scores of all patients. Patients were evaluated through the Short Form-12 questionnaire (SF-12) for quality of life assessment, the Oxford Knee Score (OKS), the Knee Society Score (KSS), and the Forgotten Joint Score-12 (FJS-12).

### 2.5. Imaging Evaluation

Preoperative and postoperative radiological evaluations were performed and stored in the hospital imaging archiving and communication system (PACS). Using a dedicated software (Carestream, Carestream Health Inc., Rochester, NY, USA) lower limb alignment was measured on long leg weight-bearing AP view, evaluating the hip-knee-ankle angle technique. The angle was measured considering the intercept point of the femoral (from the center of the femoral head through the tibial midpoint) and tibial (from the tibial midpoint to the center of the tibiotalar joint) mechanical axes [8,9]. The Knee Society Radiographic Evaluation System and Methodology protocol were used to assess the coronal alignment of the femoral and tibial component on the weight-bearing anteroposterior (AP) view. The femoral component angle is the one between the anatomic axis of the femoral shaft and distal femoral component surface and the tibial one between the baseplate and the tibia’s mechanical axis [10].

### 2.6. Statistical Analysis

All variables’ distribution was checked for normality by the Shapiro-Wilk test. For those variables for which it was applicable, missing data were addressed by the imputation of the mean. Continuous, normally distributed series are expressed as mean and standard deviation (SD); for these, longitudinal and intergroup comparison was performed by Student’s *t*-test. Series of discrete measures are expressed by median and range, longitudinal and intergroup comparison at follow-up was carried out through the Wilcoxon rank-sum test and Mann-Whitney Test. Eventually, binomial measures were compared through the Chi-square to assess the discrepancy between groups. Per convention, the significance threshold was set with alpha = 0.05. Data analysis was conducted with STATA software (Ver. 12, Stata Corp., College Station, TX, USA).

## 3. Results

According to inclusion and exclusion criteria 42 patients (18 men/24 women) that underwent to Navigated TKA (NAVI group) and 61 patients (23 men/38 women) that underwent to Conventional TKA (CONV group) were identified and included. No statistically significant difference in terms of age at surgery and BMI was detected. Mean follow-up was 3.05 years for the NAVI group and 2.99 years for CONV. Demographic parameters divided per group are summarized in Table 1.

### 3.1. Perioperative Results

Following perioperative parameters were analyzed: surgical time, intraoperative blood loss, length of hospital stays, and complications. Analyzing surgical reports, a statistically significant higher surgical time was registered for the NAVI group (86.6 ± 5.26 vs. 62.5 ± 6.94, *p* < 0.001). Although NAVI had a lower intraoperative blood loss than the control group (193.6 ± 57 vs. 235 ± 55.9, *p* 0.330), no statistically significant difference was found. Also, the length of hospital stay analysis did not show any difference between the groups. Two patients, one for each group, underwent revision surgery due to PJI (Periprosthetic Joint Infection) and aseptic loosening. One patient from the NAVI group received patellar replacement at 2.6 years postoperative because of AKP (Anterior Knee Pain). Two patients, one for each group, died for independent causes during the follow-up period. Perioperative parameters are summarized in Table 2.

### 3.2. Functional Results

**Figure 1:**
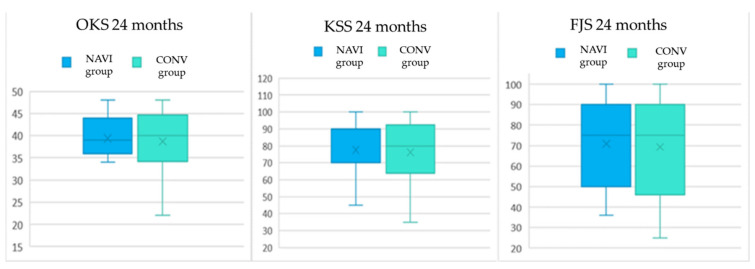
Box Plot of postoperative 24 m clinical scores.

Preoperative and two years minimum follow-up scores were analyzed. As expected, the intragroup analysis showed a statistically significant increase comparing preoperative with minimum two years follow-up value for all three scores administered (*p* < 0.001 for all three comparisons). Preoperative intergroup analysis showed a statistically significant difference in SF-12 (*p* = 0.002) in favor of the CONV group. The postoperative intergroup analysis reported no statistically significant difference between NAVI and CONV groups. Clinical data are summarized in Figure 1 and Table 3.

### 3.3. Radiological Results

**Figure 2:**
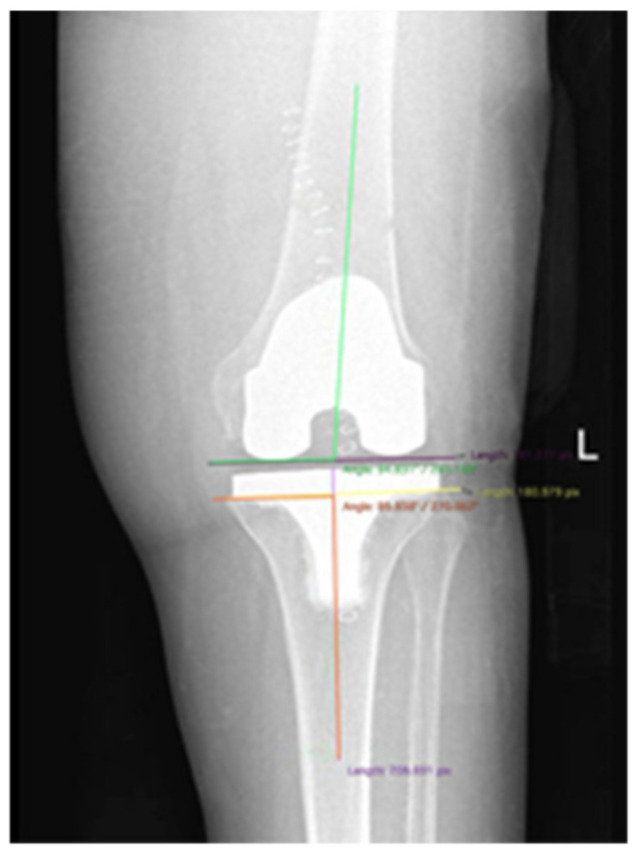
Coronal alignment measurement method for the femoral and tibial component.

**Figure 3:**
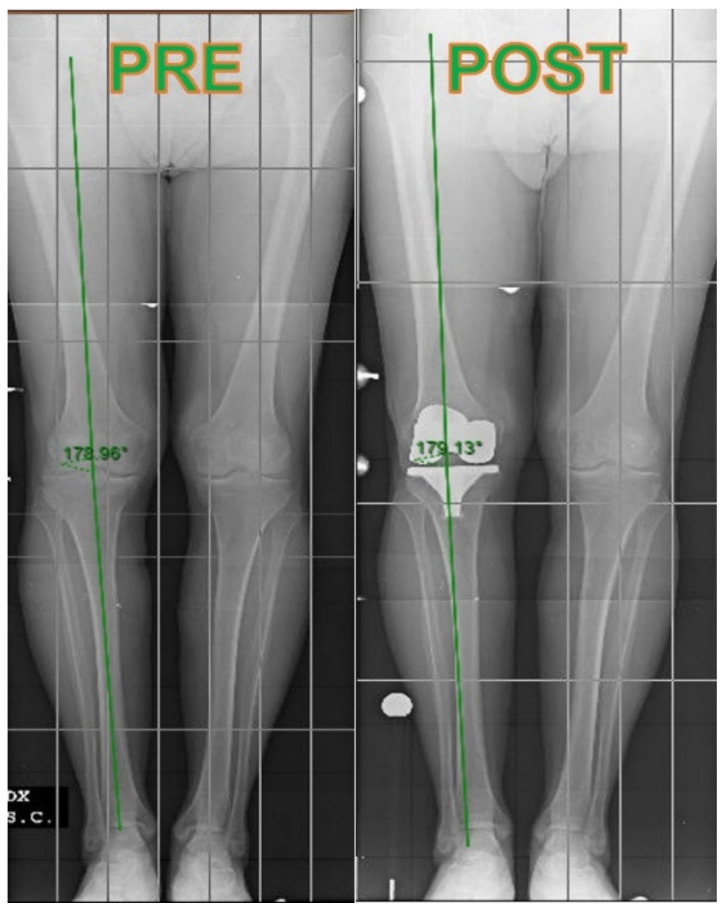
Pre and postoperative lower limb mechanical alignment.

Lower limb alignment and component positioning were measured and compared between groups. The femoral and tibial component coronal alignment (Figure 2) was measured on standard AP weight-bearing X-ray, while pre and postoperative lower limb mechanical alignment (Figure 3) was measured on full-length AP weight-bearing X-ray. The average preoperative mechanical alignment angle was 4.28° ± 6.181° varus for the NAVI group and 4.15° ± 4.524° varus for the CONV group. Postoperative lower limb alignment showed a 0.44° ± 2.408° varus in the NAVI group and 0.41° ± 2.224° varus in the CONV group. Five patients in the NAVI group and six in the CONV group presented greater than or equal to 3° of mechanical malalignment and were considered outliers. The femoral component’s coronal alignment resulted in 4.1° ± 2° valgus in the NAVI group and 3.9° ± 2° valgus in the CONV group. A slightly neutral tibial component’s coronal alignment of 0.03° ± 0.6° varus for NAVI group and of 0.23° ± 1° varus for CONV group was measured. Statistical analysis showed no significant difference between groups in any variable. Comparison data are summarized in Table 4.

## 4. Discussion

Aseptic loosening together with PJI represent the most frequent causes of TKA revisions. Mechanical malalignment with consecutive abnormal polyethylene wear is frequently at the basis of early aseptic loosening [11]. Although knee arthroplasty provides a durable long-term result, malalignment may lead to decreased prosthetic components’ survival [12]. Positive outcome and long term survival of the implant are strictly associated with component orientation, soft tissue balance, and, more importantly, the correct restoration of leg axis, that needs to achieve the desired range of ±3° of varus/valgus in the coronal plane [4,13]. Alignment is conventionally controlled with the aid of extramedullary or intramedullary alignment guides, but a proper lower limb mechanical alignment is obtained in only 60% to 80% of implants performed with a conventional technique [14]. Another cohort study highlighted a malalignment greater than 3° in 30% of conventional TKA, especially between less experienced surgeons [15]. Several computer navigation systems have been developed to achieve optimal alignment [5,16]. It has been shown that navigation in TKA improves bone resection accuracy, component coronal alignment, consequently leg alignment, and reduces blood loss compared to conventional techniques [17]. To the best of our knowledge a recent radiological study by Hannan et al. [18], performed on the same system [14], demonstrated a high concordance between intraoperative and postoperative measures performed with Perth CT protocol [19]. In the current investigation, the NAVI group reported a statistically significant higher surgical time among perioperative parameters, probably due to calibration phase and intraoperative adjustment procedures. A lower intraoperative blood loss (without a statistical significance) was found in the computer-assisted group, probably because of the absence of intramedullary alignment guide insertion. Implant survival analysis did not show differences between groups during the follow-up period. Both groups reached statistically significant higher clinical scores compared to baseline, demonstrating a treatment success. Intergroup analysis showed that implants performed with computer assistance had no postoperative difference with the conventional group using the same implant. According to radiologic analysis, the computer-assisted group showed no inferiority compared with the conventional group. After surgery, the lower limb obtained a neutral alignment and the implant a correct component position both in the tibial and the femoral side in both groups. Moreover, no difference in patients with lower limb mechanical malalignment greater than or equal to 3°, considered outliers, was found. From statistical analysis, no significant intergroup differences were found. Considering the short follow-up, the present study results agree with the current literature, and cohort analysis will be carried on for medium and long-term results. Moreover, CAS surgery should also be considered an essential training tool for residents and fellow interested in adult reconstruction [20]. Although not representing part of the present study, it could be a purpose for future research in this field. In an earlier meta-analysis, computer-navigated knee arthroplasty has been proven to result in fewer radiographic mechanical alignment outliers and better femoral and tibial component placement than conventional [21]. Hetaimish et al. [16] found that 30.1% of patients treated with conventional TKA had a deviation in the coronal plate greater than 3° from neutral versus 12.8% in patients undergoing navigated surgery. Keyes et al. [22] reported an optimal mechanical alignment in 65% of the navigated prosthesis, compared to 39% in conventional. Brin et al. [23] found an 80% reduction in the number of outliers. In one of the most recent meta-analyses, Rhee et al. [24] confirmed a higher accuracy in component positioning using CAS, but they found no significant difference in implant survival, clinical and functional outcomes than conventional TKA. Nowadays, the literature still debated the navigated technique’s superiority in clinical and functional outcomes and life quality. Another recent systematic review and meta-analysis [25], published in 2020, reported a slight improvement in functional outcome using CAS, considering studies with 4 or 5 years of follow-up. The routine role of navigated TKA is still questioned by the costs, the additional operating time, the increased training, the potential for new and increased complications, and the lack of reproducible evidence [26]. Nowadays, while in the United Kingdom (UK) and the United States of America (USA), only 3–5% of TKA performed annually utilize CAS navigation, while in Australia, the rate of CAS navigation has increased from 2.4% in 2003 to 30.8% in 2016 [26]. Therefore, although mechanical axis accuracy and component positioning have been shown to improve, CAS superiority in functional outcome is still controversial, and it is unclear if these improvements have translated into improved knee function and quality of life. The main limitations of the present study are its retrospective nature, the relatively small sample size, and the short follow-up, for the type of procedure. This study also presented several strengths. Two experienced adult reconstruction surgeons performed all surgical procedures. Two independent investigators not involved in the surgical procedures examined the patients at follow-up, and another one performed radiologic measurements. All data were analyzed in blind to the surgical group. All patients in both groups were treated with the same implant type.

## 5. Conclusions

The present study showed that there was no difference between CAS arthroplasty and conventional technique concerning component positioning and functional recovery. CAS resulted accurate and able to receive an intraoperative adjustment. Future controlled studies on the same system, with a randomization process, larger sample size, and a longer follow-up are needed.

## Figures and Tables

**Table 1:** Demographic parameters.

Demographic Parameters	Navi	Conv	*p*
Sex (Men/Women)	18/24	23/38	
Mean age at surgery (yrs.)	71.9 ± 6.16	71.5 ± 6.24	0.658
BMI (Kg/m^2^)	31.4 ± 4.95	29.1 ± 1.65	0.984
ASA score (Median)	2	2	
Follow-up (Months)	36.3 ± 4.92	35.88 ± 6.96	

**Table 2:** Perioperative parameters.

Perioperative Parameters	Navi	Conv	*p*
Surgical Time (min)	86.6 ± 5.26	62.5 ± 6.94	*p* < 0.001
Blood loss (cc)	193.6 ± 57	235 ± 55.9	0.330
Length of stay (days)	3.8 ± 2.56	3.1 ± 2.16	0.818
Patellar replacement (%)	92.9%	90.2%	
Complication	1 PJI 1 Patellar replacement	1 Aseptic loosening	

**Table 3:** Clinical parameters.

Clinical Parameters	Navi	Conv	*p*
SF-12 MCS pre	45.69 ± 8.72	47.68 ± 12.16	0.238
SF-12 MCS 24 m	56.9 ± 6.72	55.74 ± 10.66	0.272
SF-12 PCS pre	25.49 ± 7.25	21.43 ± 4.09	0.002
SF-12 PCS 24 m	44.55 ± 8.03	43.08 ± 10.60	0.193
OKS pre	15.61 ± 5.06	16.97 ± 4.27	0.102
OKS 24 m	39.38 ± 6.31	38.73 ± 7.47	0.339
KSS Knee pre	46.7 ± 13.31	44 ± 11.44	0.335
KSS Knee 24 m	77.54 ± 20.56	76.25 ± 19.59	0.774
FJS 24 m	70.88 ± 21.49	69.32 ± 24.50	0.380

**Table 4:** Radiological parameters.

Radiological Parameters		Varus (+) Valgus (−) PRE	Varus (+) Valgus (−) POST	Femoral Coronal Alignment	Tibial Coronal Alignment
CONV	Mean	4.15	0.41	93.98	89.77
	N	61	61	61	61
	Std. Deviation	4.52	2.22	2.01	1.09
	Minimum	−7	−4	87	88
	Maximum	10	5	98	93
NAVI	Mean	4.28	0.44	94.11	89.97
	N	42	42	42	42
	Std. Deviation	6.18	2.40	2.06	0.65
	Minimum	−10	−4	89	89
	Maximum	15	5	98	91
Total	Mean	4.26	0.43	94.04	89.86
	N	103	103	103	103
	Std. Deviation	5.29	2.29	2.02	0.92
	Minimum	−10	−4	87	88
	Maximum	15	5	98	93

## Data Availability

Dataset used for study analysis are available upon motivated request to the corresponding author.
